# A guide to reducing adverse outcomes in rabbit models of sciatic nerve injury

**DOI:** 10.1186/s42826-021-00085-1

**Published:** 2021-05-17

**Authors:** Elisabeth Orozco, Koichi Masuda, Sameer B. Shah

**Affiliations:** 1 Research Division, VA San Diego Healthcare System, San Diego, CA USA; 2 Department of Orthopaedic Surgery, University of California, San Diego, La Jolla, CA USA; 3 Department of Bioengineering, University of California, San Diego, La Jolla, CA USA

**Keywords:** Peripheral nerve, Nerve injury, Rabbit, Bandaging, Pressure ulcer, Elizabethan collars

## Abstract

**Background:**

Peripheral nerve damage can have debilitating consequences. Rabbit sciatic nerve transection models allow the effective evaluation of surgical repair strategies for large nerve gaps. Despite advantages in size, ease of handling, and functional utility, rabbits can suffer from a number of side effects that affect animal welfare and the quality of scientific inquiry. Such side-effects, which include pressure ulcers and traumatic damage to the foot, are primarily a consequence of insensitivity of the distal hindlimb following sciatic nerve injury. In this study, we present a number of methodologies for identifying, treating, and preventing unintended adverse effects in rabbit sciatic nerve injury models.

**Results:**

First, we categorize pressure ulcers according to their severity and describe the deployment of a padded bandaging technique to enable ulcer healing. We also introduce a proactive bandaging approach to reduce the likelihood of pressure ulcer formation. Second, we define phenotypes that distinguish between foot injuries resulting from self-mutilation (autotomy) from those caused by incidental traumatic injury secondary to sensori-motor damage. Finally, we detail an effective strategy to reduce the usage of Elizabethan collars; through a gradual weaning protocol, their usefulness in preventing autotomy is retained, while their propensity to impede rabbit grooming and cause abrasion-injury to the neck region is minimized.

**Conclusions:**

We suggest that application of these methods offer a practical and systematic approach to avoid adverse side effects associated with rabbit sciatic nerve damage, enabling improved animal welfare and scientific outcomes in a powerful nerve injury model.

## Background

Peripheral nerve damage is a serious injury that can lead to debilitating sensorimotor dysfunction and prolonged pain. Most traumatic nerve injuries in the general population are due to automobile accidents and household accidents [1, 2], while the vast majority of traumatic nerve injuries in military settings are due to explosives and gunshot wounds [3, 4]. A number of mammalian models varying in size and anatomy have been used to understand nerve function, probe mechanisms of nerve injury, and evaluate strategies for repair and regeneration, including mice, rats, hamsters, guinea pigs, rabbits, pigs, sheep, cats, dogs, and non-human primates [5–12]. Rats are the predominant model for hypothesis testing related to peripheral nerve injury, degeneration, and repair, as they are docile, cost effective and amenable to microsurgical repair of a reasonable gap size (typically < 15 mm for the rat sciatic nerve) [13, 14]. Mouse nerves are not of sufficient size for evaluating gap-repair strategies, but are frequently deployed to understand mechanisms of nerve damage and recovery, given the relative ease and scientific power of their genetic manipulation [15, 16]. After proof of concept testing in smaller animals, larger animals are used to test strategies for repairing gaps more comparable in size to those in humans. While feline, canine, and ovine models have been used historically to investigate large nerve gaps [5–8, 10], unique housing and husbandry requirements as well as increasing ethical scrutiny have reduced the frequency of their usage. Porcine and non-human primate models represent the gold-standard with respect to simulating the size, anatomy and physiology of human nerves, and are therefore used in translational or late-stage pre-clinical research [10–12]. However, these models are costly and require highly specialized laboratory facilities and veterinary care; they are thus comparatively impractical.

Therefore, rabbits are often used as a non-rodent model for larger nerve injuries [17]. Among the peripheral nerves of rabbits, though models of facial and upper extremity nerve injury are not uncommon [18–20], its substantial length (which easily accommodates gaps exceeding 3–4 cm) and ready access render the sciatic nerve the most common choice for injury and repair. There are multiple additional benefits to using rabbits as a surgical model, including ease of handling and cost-effectiveness compared to larger animal models, in vivo functionality, biocompatibility/safety, and clinical relevance/efficacy [21]. Although rabbits are a useful research model to study nerve injury, rabbit studies can suffer from a number of adverse side effects. The most prominent among these adverse effects result from the lack of sensory feedback from the insensate distal hindlimb resulting from sciatic nerve injury. The combination of this insensitivity and consequent imposed trauma during otherwise innocuous foot stamping can lead to the development of pressure ulcers on the rabbit hocks [17]. This issue is compounded by the facts that rabbits have minimal sub-dermal padding at the heel, and the normally thick layer of fur cushioning the soles of their feet is often compromised after nerve injury. Further, dysesthesia and loss of muscle function following injury or during early or incomplete regeneration may result in foot dragging or self-mutilation (autotomy), requiring amputation [17, 22].

These complications have negative consequences, both with respect to animal welfare as well as the quality of evaluating strategies for nerve repair. Logistically, unintended complications can lead to extensive and costly animal care, including daily veterinary care and additional surgical intervention. Severe injuries can also lead to unnecessary infection, animal pain, and distress, therefore requiring euthanasia for ethical reasons at an earlier than desired time point. Finally, complications can also impact the ability to collect data or data quality. Depending on the severity of the pressure ulcer, foot trauma, autotomy, or other unintended adverse effect, sensori-motor, gait, vascular, and regenerative outcomes may be confounded or compromised.

On the other hand, reducing adverse outcomes in rabbit models can lead to a reduction in animal care burden, improvement in the quality of scientific evaluation, improved cost and time efficiency, and more humane treatment of animal subjects. In this study, towards such goals, we provide insight into unintended complications in rabbit sciatic nerve injury models and introduce methodology to prevent and/or treat such complications. We hypothesize that our methods lead to a reduction of adverse pressure ulceration and phalangeal injury associated with nerve injury, enabling unconfounded experimental data collection and humane post-operative care.

## Results

### Characterization of pressure ulcer severity

**Fig. 1:**
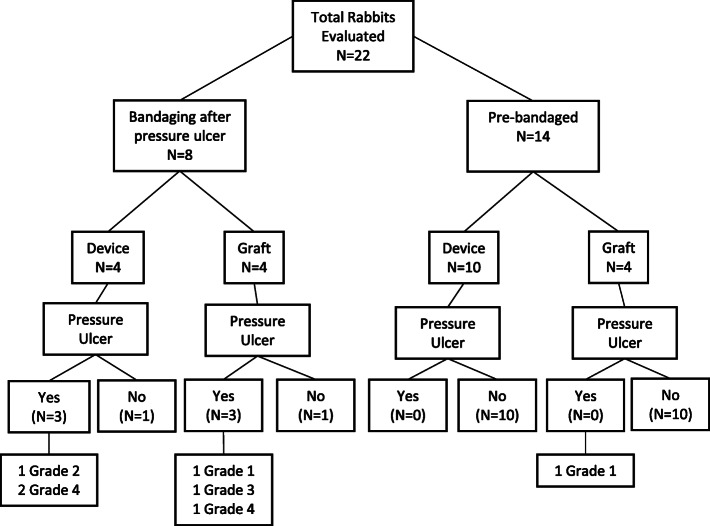
Summary of evaluated animals and pressure ulcer classification

**Fig. 2:**
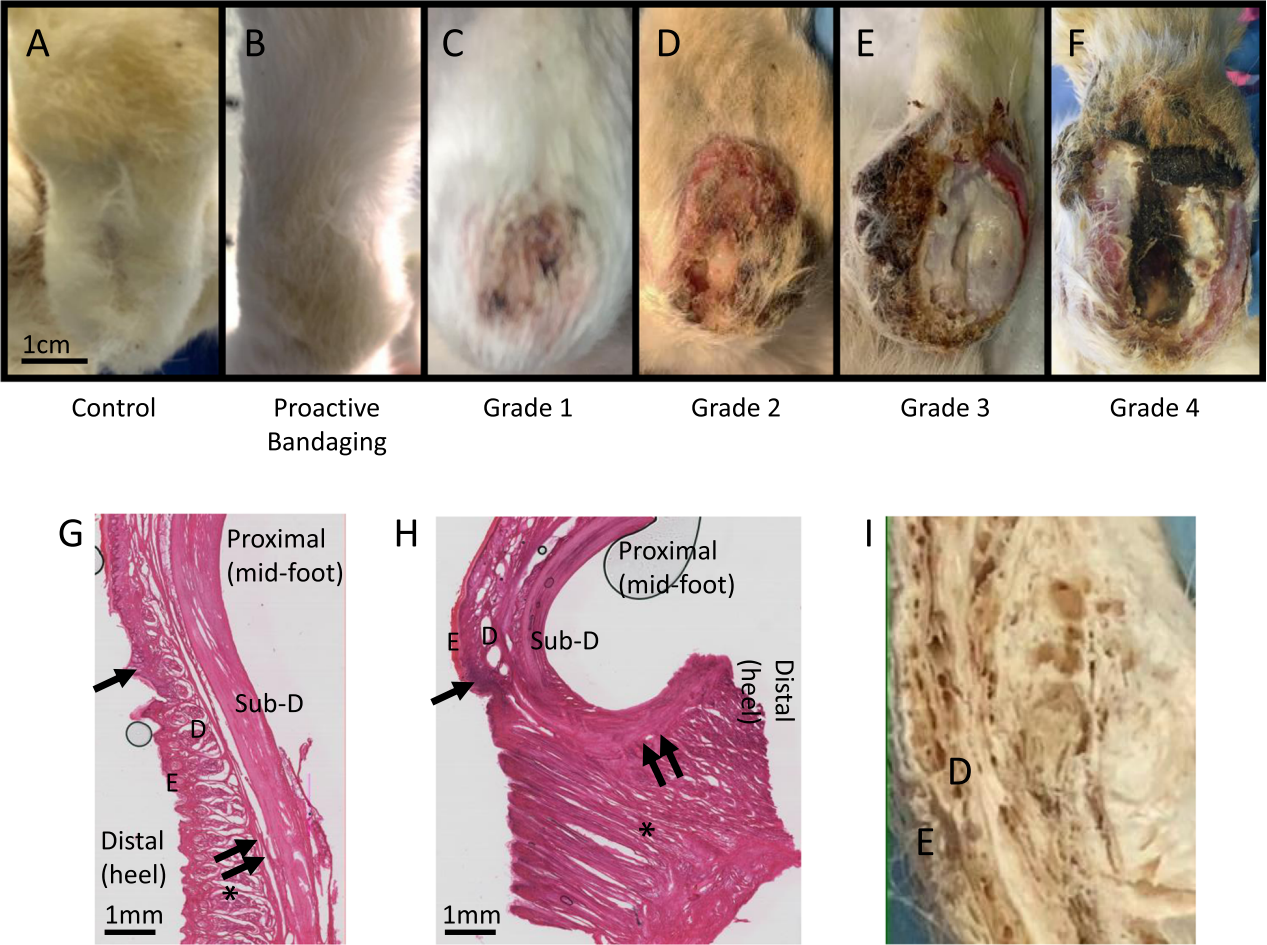
Severity of pressure ulcers. Healthy hindpaw seen on (**a**) control and (**b**) proactively bandaged hindpaw. Pressure ulcers were seen on the heel within 1.5 weeks of nerve injury without standard bandaging. **c** Grade 1 pressure ulcers, seen within 1–2 weeks of nerve injury and infrequently when standard bandaging was implemented proactively. **d-f** Grades 2–4 pressure ulcers were seen when treatment was implemented within 2–3 weeks after nerve injury. **g-h** Eosin labeling in sagittal sections of (**g**) Contralateral control paw and (**h**) ipsilateral paw that was denervated and bandaged for 4 months, revealed no visible ulceration. Single and double arrows denote corresponding locations along length of paw in control and denervated limbs. Despite considerable scar formation and connective tissue remodeling in the heel (region indicated by *), which is concurrent with contracture (resulting in high curvature of denervated paw), no breaks in the integrity of the skin were observed with bandaging. E: Epidermis; D: Dermis; Sub-D: Subdermal tissue (muscle, fascia). **i** Gross morphological view (axial section) of scar deposition in the heel, resulting from prolonged denervation, analogous to * region in (H)

All animals survived through the evaluation period (Table 1, Fig. 1), during which pressure ulcers of varying severity were observed. We developed a semi-quantitative scale to score the severity of the pressure ulcer (Table 2, Fig. 2a-f). Two rabbits were categorized as Grade 1, one as Grade 2, one as Grade 3, and three as Grade 4. Fifteen rabbits had no signs of a pressure ulcer. There were no differences in the distribution of categories among autograft-treated and device-treated nerve injuries (Table 1, Fig. 1).

**Table 1:** Details of subjects included in our study

Animal ID	Pressure Sore (yes/no)	Pre-bandaged (yes/no)	Ulcer Grade	Surgery (Device/ Graft)
Animal 1	Yes + infection	No	Grade 4	Device
Animal 2	Yes + infection	No	Grade 4	Device
Animal 3	Yes	No	Grade 2	Device
Animal 4	No	No	n/a	Device
Animal 5	Yes	No	Grade 3	Graft
Animal 6	Yes	No	Grade 1	Graft
Animal 7	No	No	n/a	Graft
Animal 8	Yes	No	Grade 4	Graft
Animal 9	Yes	Yes	Grade 1	Graft
Animal 10	No	Yes	n/a	Graft
Animal 11	No	Yes	n/a	Graft
Animal 12	No	Yes	n/a	Graft
Animal 13	No	Yes	n/a	Device
Animal 14	No	Yes	n/a	Device
Animal 15	No	Yes	n/a	Device
Animal 16	No	Yes	n/a	Device
Animal 17	No	Yes	n/a	Device
Animal 18	No	Yes	n/a	Device
Animal 19	No	Yes	n/a	Device
Animal 20	No	Yes	n/a	Device
Animal 21	No	Yes	n/a	Device
Animal 22	No	Yes	n/a	Device

**Table 2:** Scale used to score the severity of pressure ulcers

Grade	Symptoms
Grade 1	Hair loss at the bottom of the foot, redness, minor skin abrasions
Grade 2	Hair loss, red skin, and break in skin, minor scab overlying break
Grade 3	Hair loss, red skin, swollen tissue, pus visible, peripheral scab formation
Grade 4	Hair loss, red skin, inflamed deep tissue and pus visible, abscess and/ or infection

### Influence of bandaging on pressure ulcer formation

There was a significant difference in pressure ulcer formation between rabbits that were pre-bandaged and rabbits that were bandaged 3 weeks post-surgery, with pre-bandaging markedly reducing the likelihood of ulcer formation (Table 1, Fig. 1; χ^2^ = 16.18, *p* < 0.05, *N* = 22, 3 degrees of freedom). Interestingly, bandaging was protective against ulceration even in the presence of substantial scar formation in the hindlimb following chronic denervation (Fig. 2g-i).

### Complications and caveats

#### Use of e-collars

**Fig. 3:**
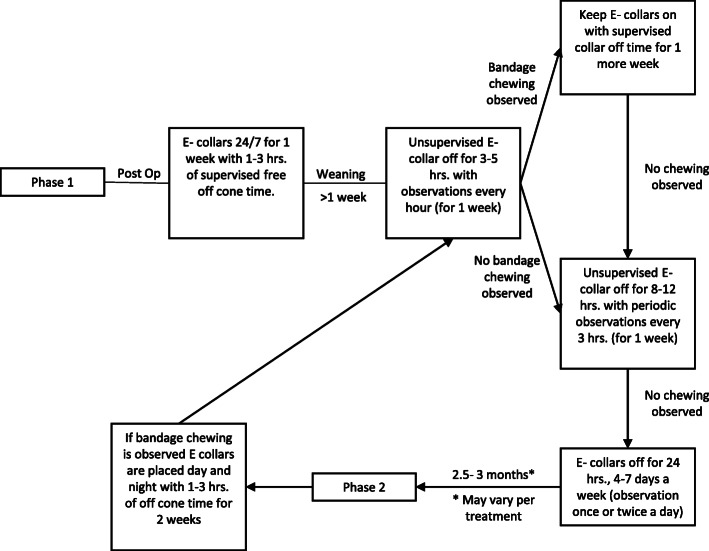
E-collar usage decision flow chart and timeline. The rationale for this flow chart is to deploy e-collars at key time points when self-mutilation may occur (immediately after surgery and during time frames associated with initially restored function), but otherwise systematically wean animals off of e-collar usage

E-collars were placed on rabbits after surgery to prevent the animal from accessing surgical incisions or implanted devices. In addition, e-collars were used 3 months after repair, when risk of self-mutilation was increased due to re-emerging sensation. We developed a flow chart to describe a strategy for gradual weaning of e-collars with liberal off-collar time, with progressively decreasing levels of supervision or observation (Fig. 3).

#### Bandaging

**Fig. 4:**
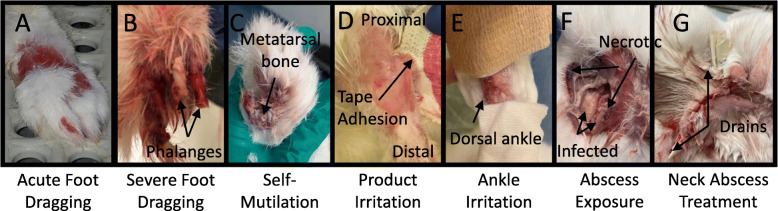
Indirect injuries associated with improper bandaging or prolonged E-collar use. **a-b** Foot dragging injuries due to unraveling of bandage ranging from (**a**) focal to (**b**) severe injury. **c-d** Distinction between (**c**) severe foot dragging and (**d**) self-mutilation. Phalanges are visibly exposed but intact due to foot dragging, while phalange has bite marks or is chewed off on self-mutilated rabbits. Self-mutilation cannot occur with an E-collar. **e** Athletic tape can lead to skin irritation and skin breakage. **f** Ankle irritation due to lack of protective gauze on the dorsal region of the ankle. **g** Neck abscess from prolonged E-collar use (right)

**Fig. 5:**
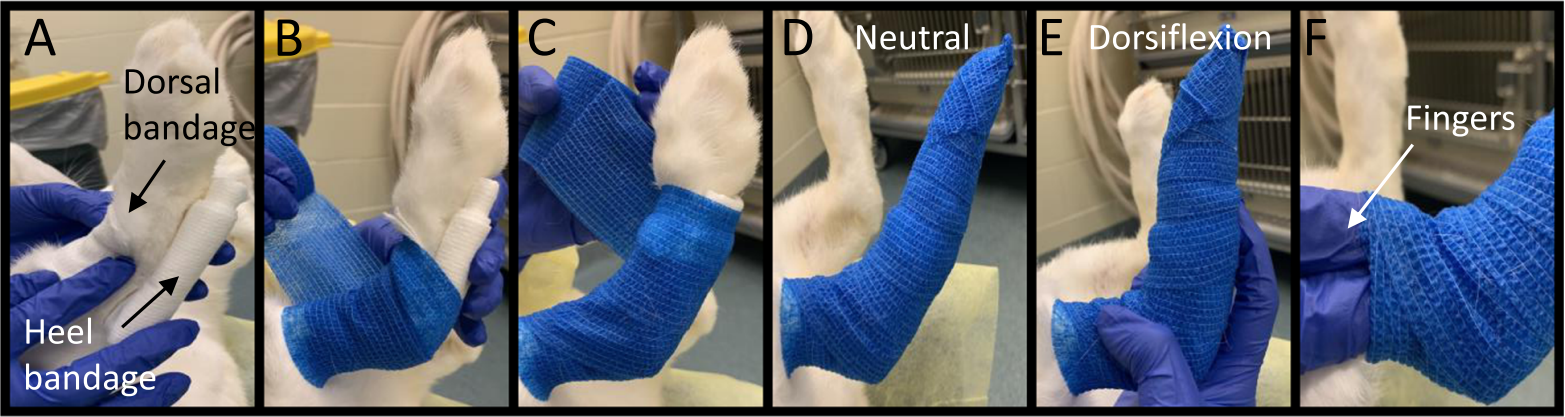
Bandaging guidelines. **a** One piece of gauze was placed on the dorsal region of the ankle and two pieces of gauze folded in half were placed on the heel. Half of the length of the bandage was used to cover above the heel while the other half was used to cover the length of the foot. **b**, **c** The adhesive bandage was wrapped twice around the entire foot. **d** Neutral configuration. Adhesive bandage was wrapped along the entire foot with gentle compression to ensure that the bandage adheres. **e** Foot is shown to dorsiflex without any issues. **f** Two fingers were placed by the opening by the ankle to check for compression

**Fig. 6:**
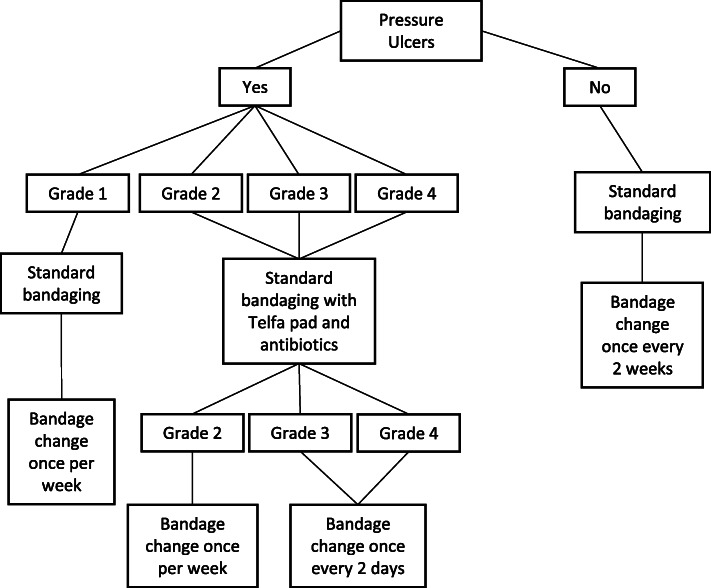
Pressure ulcer treatment decision flow chart. Even if no pressure ulcer is observed, proactive bandaging is highly encouraged to prevent pressure ulcer development. If pressure ulcer is observed, standard bandaging treatment should be implemented immediately to prevent the progression of the pressure ulcer and an infection

Foot bandaging was indeed useful for treating and preventing pressure ulcers; however, it was not uncommon for rabbits to loosen or shake off their foot bandage during movement. An adverse outcome associated with bandage removal was foot dragging (Fig. 4a-b) – distinct from self-mutilation (Fig. 4c) -- caused due to the loss of dorsiflexor muscle mass and/or lack of proprioceptive feedback, which prevents the rabbit from lifting up its injured foot. Damage resulting from foot dragging increases based on the duration over which this activity occurs. Dragging for durations of a few minutes to 2–3 h resulted in minor foot redness to more irritation and swelling, respectively. For longer durations, abrasion or breaks in the skin were observed, and in severe cases of overnight foot dragging, toe amputation was required due to severe skin loss/phalangeal exposure. Proactive bandaging, including a dorsal layer of gauze (Figs. 5 and 6) was helpful in reducing foot dragging injuries. In the event of injury, topical ointment, gauze, and self-adherent bandaging (but not athletic tape; Fig. 4d) are recommended, with suturing or amputation required for more severe injuries (Table 3). Bandaging also occasionally resulted in irritation of the dorsal aspect of the ankle (Fig. 4e); this could be mitigated with a layer of gauze.

**Table 3:** Detailed treatment protocols and additional rationale

Injury	Solution	Rationale
Pressure Ulcer	1. Debride with chlorohexidine and Neosporin. 2. If break in skin, place Telfa pad directly on wound. 3. Layer two 4 × 4 layers of gauze padding at the heel and one 2 × 2 gauze layer at the ankle. 4. Start self-adherent bandage above the ankle and work towards the toes. Check for excessive compression throughout your bandaging process. Note: Depending on the severity of the pressure ulcer, antibiotics might be necessary. Bandage should be changed every 2 days or once a week depending on the severity of the pressure ulcer. Bandage should be changed once every two weeks when there is no pressure ulcer.	Animals are insensate; therefore, they have no painful feedback preventing them from injuring themselves. Bandaging provides extra cushioning at the heel for when the animal stamps its foot. The Telfa pad prevents gauze from sticking to the wound.
Foot Dragging	Minor Irritation: 1. Place one 2 × 2 gauze layer over the affected region of the foot. Break in the Skin: 1. Remove any necrotic tissue. 2. Place one Telfa pad directly on wound. 3. Place a layer of 4 × 4 gauze for padding before adhesive bandage. 4. Wrap self-adherent bandage as above. 5. Antibiotic treatment for 7 days. Note: Depending on severity of the injury, suturing or amputation of toes might be required. *No E-collar placement is necessary.* Bandage should be changed once a week.	The wound should be shielded from additional damage. Removal of necrotic tissue is necessary to allow healthy tissue to grow. Suturing is only necessary when bone is exposed. If a layer of muscle is exposed with no bone exposure, suturing is not required. Amputation is required if phalanges are exposed.
Self-Mutilation	1. Debride and sterilize injury site. 2. Toe amputation at the metacarpal will be necessary unless there is enough healthy tissue to close the opening. 3. Rinse with saline before suture closing. 4. Apply Triple antibiotic and Telfa pad on the wound. 5. Place one 4 × 4 gauze layer on top of the Telfa pad. 6. Wrap self-adherent bandage as above. 7. Place E-collar immediately, with periodic supervised off-collar time.	The intent is to clean and close the wound, and shield wound from additional damage. E-collars are placed to prevent further self-mutilation. If animals do not show indications of additional self-mutilation during supervised off-collar time, E-collar usage may be reduced through a systematic weaning process.
Neck Abscess	1. Excise necrotic tissue and flanking margins of healthy tissue. 2. Either leave incision open to drain on its own or implant drains. 3. Apply Triple antibiotic and one Telfa pad. 4. Place one layer of undercast padding. 5. Place two layers of 4 × 4 gauze *only* at the injury site. 6. Place one stockinette around the neck. 7. Wrap a loose self-adherent bandage around the neck. Place two fingers between padding and neck to avoid over- compression. Note: BupSR must be given for pain management. NSAIDs are unlikely to sufficiently alleviate pain. With every bandage change, slowly shift drains side to side to help the pus drain. Bandage should be changed daily until draining subsides. Suturing of a neck wound may be required based on the depth of the wound, per consultation with veterinary staff.	Bandaging both shields the wound from additional trauma and provides a distraction for the rabbit, so as to minimize grooming/pawing of the wound area. For the latter, a loose bandage that slips off of the wound wsa observed to be favorable compared to an absence of bandaging. The stockinette is placed to prevent the gauze from rolling out from the bandage and to keep the adhesive bandage in place.
Dorsal Ankle Abrasion or Injury	1. Apply one layer of 2 × 2 gauze 2. Wrap self-adherent bandage as above. Note: If possible, try to keep the fur on the area (do not shave – trimming of hair with scissors will suffice and preserve some padding). Bandages should be continuously wrapped, to reduce likelihood of unraveling.	The dorsal ankle is the main pressure point of the bandage during typical paw positioning, and is a generally delicate area. There is less likelihood of stamping or dragging trauma in this region, but gauze provides some padding in a mobile and flexible area.
Product Irritation	1. Do not use athletic tape (e.g., Elastikon) to secure self-adherent bandage. 2. Gauze should not directly contact an open wound.	Athletic tape is extremely adhesive, while rabbit skin is very fragile; therefore, removal of the athletic tape has the potential to tear the rabbit skin (see images). Telfa pads provide a suitable protective barrier between gauze and open wound.

## Discussion

### Bandaging reduces pressure ulcer formation and enables healing

Our data indicate the clear benefit of proactive bandaging. We therefore recommend that bandaging can and should be implemented immediately after nerve injury surgery, to prevent the development of pressure ulcers, irrespective of anticipated regenerative outcome. Proactive bandaging was also helpful in reducing foot dragging injuries and other traumatic injuries to the foot. Several rationale were considered in developing our bandaging strategy (Table 3, Figs. 5-6). Key elements of this strategy include: 1) Cushioning the heel/hock with gauze to prevent direct impact to the bone of the animal, as skin and padding is thin in this region. 2) Telfa pad in direct contact with the wound to prevent discharge adhesion to gauze; 3) adhesive bandage wrapped around the toes, to prevent adverse effects from foot dragging; 4) sufficiently loose bandaging to reduce constriction; this accounts for an inability to visualize toes, which is typically recommended for ankle bandaging (e.g., ankle sprain).

Bandaging recommendations are generally consistent with recommendations of Farinas et al., including complete bandaging from above the ankle around the entire foot, as well as additional padding at the heel [17]. Key technical differences include our usage of Telfa pad and gauze rather than a custom-fabricated piece of foam and undercast padding as cushioning; both methods would presumably achieve the same purpose. On the other hand, there are several differences in our findings. Reported rates of self-mutilation in our model were lower in our cohorts, possibly due to differences in e-collaring procedures, or because we distinguished between foot-dragging injuries (which could occur with an e-collar on and did not result in bite-marks on bandaging or tissue) vs. self-mutilation (which could only result when e-collars were removed). Prudent e-collar usage and bandaging reduced the incidence of both. Thus, given its many advantages in scale and physiology, we advocate the continued usage of a rabbit nerve injury model, with appropriate precautions.

### Reducing other complications

The benefits of e-collaring to protect surgical incisions and prevent self-mutilation underlie typical recommendations for almost constant use of e-collars with rabbit nerve injury models. On the other hand, long periods of time with e-collars can lead to a number of complications. Phenotypes such as a dull or disheveled coat, buildup of mildew or other dirt in the vicinity of the e-collar, porphyrin build up, crusting, and irritation surrounding the eyes, and in the most severe conditions, neck abscesses (Fig. 4f-g). The latter is a considerable challenge and may be limited by padded donut collars [17]; care should be taken to identify durable materials that will not fragment and be subsequently ingested. Based on attempting to balance the pros and cons of e-collar usage, careful observation of each subject can be helpful. Distinguishing between normal grooming behavior, tendency for self-mutilation, and adverse consequences of foot dragging can help guide a decision of whether and for how-long to limit e-collar use. Should a decision be made to minimize e-collar use, a gradual weaning of e-collars with liberal off-collar time under supervision or regular observation can be helpful (Fig. 3).

## Conclusions

In our study we have provided insight on a variety of unintended complications that we encountered when working with a rabbit nerve injury model, including the formation of pressure ulcers and guidance on e-collar usage. We have provided a detailed treatment plan to treat all grades of pressure ulcers, as well as a successful preventative pressure ulcer plan. Timing of intervention and the type of treatment influences the progression or prevention of pressure ulcers following nerve injury. In particular, our data suggest that proper foot bandaging immediately after surgery in combination with daily observation prevent severe pressure ulceration. However, even in the absence of such preventative measures, pressure ulcers from Grade 1–3 are reversible if treatment is implemented immediately. A Grade 4 ulcer can be reversible if proper treatment is implemented and there are no signs of infection. When combined with careful observation, we also advocate considerable time for rabbits free of e-collar usage. Under such guidance, rabbits remain a humane and powerful non-rodent model of peripheral nerve injury.

## Methods

### Animal model and surgery

All procedures were approved by VASDHS and UCSD Institutional Animal Care and Use Committees (IACUC protocols #A15–011 and #S08258, respectively). Twenty-two female New Zealand white rabbits were purchased through Western Oregon Rabbit Co. (Philomath, Oregon), and single-housed. Operations were performed on 5-month-old rabbits, weighing 3.2-4 kg.

Prior to intubation, rabbits were given Ketamine (35 mg/kg) and Xylazine (5 mg/kg) subcutaneously as a short-term anesthetic. They were also provided a one-time subcutaneous dose of slow-release Buprenorphine (BupSR, 0.15 mg/kg) for pain management and a daily subcutaneous dose of Baytril (5 mg/kg) for 7 days as an antibiotic. Upon intubation, animals were transitioned to 1–5% isoflurane for anesthesia via inhalation. Vitals were monitored via pulse oximeter and a thermometer and depth of anesthesia was also monitored qualitatively by tracking the animal’s reflexes. Body temperature was maintained at 37 °C via a water-circulating heating pad for the duration of the surgery. After surgery, rabbits were placed in a Bair hugger (3 M, Canada) until full recovery before being returned to their housing.

Animals subject to two different sciatic nerve repair strategies, an autologous graft (autograft) or a nerve lengthening device, were used to evaluate adverse outcomes at early time points after sciatic nerve injury (Table 1, Fig. 1). For both groups, the length of the sciatic nerve was accessed by a single incision through the dorsal region of the upper hindlimb of the rabbit. In order to decrease damage to overlying muscles, separation of the two heads of the biceps femoris muscles was performed by blunt dissection, and surgical retractors used to provide adequate exposure of the sciatic nerve. A 20 mm nerve segment was sharply transected using a #11 surgical blade. Both repair strategies were performed within the exposed nerve bed. For the autograft group, the 20 mm segment was then reversed (proximal end oriented distally) and reattached using four 8–0 polyglycolic acid (PGA) sutures. In the second group, after the sciatic nerve was exposed, an experimental nerve lengthening device was implanted. Details on this device are modified from those presented elsewhere [23, 24]. Briefly, a stainless steel backbone was secured to the femur, just proximal to the knee joint via 1.4 mm × 10 mm titanium orthodontic screws. Nerve stumps were secured to the device by means of proximal and distal nerve cuffs 3D-printed using Visijet M3 Crystal polymer [24], which allowed the proximal stump to be pulled towards the distal stump along the backbone, by means of an externalized stainless steel guidewire exiting laterally, mid-thigh. After the device was implanted, it was then surrounded in an anti-fibrotic 0.2% alginate solution gelated within the nerve bed by the addition of 102 mM calcium chloride [25–27]. The proximal stump was then actuated 2 mm (< 10%) per day by manually pulling the guidewire for 10–15 days at a slow strain rate. After 2 weeks, the device was extracted and an end to end repair was performed using 8–0 PGA sutures. Data from the graft and device repairs were pooled, as there were no differences between the two groups with respect to adverse outcomes of the hindpaw (Table 1, Fig. 1) [3].

Beyond these animals, a single rabbit was subjected to a sciatic nerve injury as above for histological analysis. Autograft repair was unsuccessful (i.e., chronic denervation), and the animal was perfusion-fixed via the abdominal aorta after 4 months of “recovery.”

### Post-operative care

Rabbits were monitored daily after surgery for 1 week or until device explantation. Rabbits were monitored weekly for up to 6 months. At their terminal time point, rabbits were euthanized via an intravenous dose of Euthasol (0.22 ml/kg). Pressure ulcer outcomes in this study were evaluated at time points < 1 month after injury, and so are independent of any regenerative outcomes.

### Evaluation of pressure ulcer severity

Digital images were taken of rabbit paws to evaluate the severity of pressure ulcer formation. Scoring was performed based on foot pad fur loss, whether there was a break on the skin, swelling of the ankle, ulcer development, and if there were any signs of an infection (Table 2, Fig. 2).

### Bandaging

One cohort of eight rabbits was bandaged a few weeks after their initial surgery, after adverse symptoms emerged, while another cohort of fourteen rabbits were preemptively bandaged immediately after surgery. Among those bandaged at a delay, Grade 1 and Grade 2 rabbits were bandaged 1.5 weeks after their repair surgery, while Grade 3 and 4 rabbits were bandaged approximately 3 weeks after their surgery. A detailed protocol for bandaging is provided (Table 3, Figs. 5-6). Briefly, open wounds were disinfected with chlorhexidine and topically coated with Neomycin/polymyxin B/bacitracin (triple antibiotic) ointment. Telfa pads, when required, were placed in direct contact with the wound, to prevent fluid adhesion to the gauze. Folded gauze provided extra cushion at the heel of the rabbit. Self-adherent bandages were wrapped around the entire foot, including the toes. Two fingers were inserted just proximal to the ankle as confirmation that bandages did not over-compress. Athletic tape (e.g., Elastikon) was not used to secure elastic bandages, as pilot usage resulted in skin irritation and/or breakage.

### Elizabethan collars

Elizabethan collars (a.k.a., e-collars or collars; 59–6428, Harvard Apparatus, Holliston, MA) were placed on the animals to prevent the animals from chewing on their surgical incision or self-mutilating. Collars were deployed in two phases. Immediately after surgery, collars were placed for 1 week or until device explantation with 1 h of daily off-collar free time under supervision. Animals were then weaned off of the collars, with progressively increasing off-collar time (Fig. 3), with “good behavior,” indicated by no apparent chewing of bandage or incision site, rewarded with additional off collar time. Any evidence of chewing resulted in full-time collar usage with limited supervised off-collar time, at which point the weaning process was re-initiated. After ~ 2.5–3 months, at which time point sensation appeared to recover (indicated by changes in stance or additional attempted grooming of injured paw), e-collars were replaced on any animals observed to exhibit behavior suggestive of self-mutilation (e.g., chewing on bandage, excessive attention to hindpaw, and nibbling on toes even under supervision,). Weaning was performed as above. Details on E-collar usage are provided in Table 3 and Fig. 3.

### Neck abscess

Neck abscesses emerging due to irritation from e-collars were treated surgically. Rabbits were provided Ketamine (5 mg/kg), Baytril (5 mg/kg), and BupSR (.15 mg/kg) before surgery as above. Carprofen (an NSAID) was used for any persistent pain, per veterinary recommendation. A medial-lateral incision was made in the location of the abscess, the wound disinfected and necrotic tissue as well as margins of flanking healthy tissue excised. For larger abscesses, drains were sutured at the edge of each incision prior to closure (1 drain for every ~ 4 cm of incision length). Drains were gently shifted side to side during bandage changes, to help drain the highly viscous discharge. Triple antibiotic, one Telfa pad, and two 4 × 4 gauze were overlaid upon the incision. A stockinette and loose bandage were placed around the neck to stabilize padding and to prevent the animal from chewing its incision. To ensure that air flow was not obstructed, two fingers could be inserted between the bandage and skin. Bandaging was continued for 1 week after wound sealing. Additional details are provided in Table 3.

### Amputation

Amputations were required due to severe instances of foot dragging or self-mutilation. Wounds were cleaned and sterilized using chlorhexidine and 90% ethanol. An incision was made along the length of the toe, sufficient to expose the metacarpals. The damaged bone was transected at the distal metacarpal using bone cutting scissors. Upon removal of the phalanges, the incision was rinsed, overlying muscle and fascia closed with interrupted 2–0 PGA suture, and skin closed with 4–0 PGA suture. Triple antibiotic, one Telfa pad, and one 4 × 4 gauze were overlaid upon the incision, and foot bandaging was performed as described above.

### Histology

Eosin labeling was performed on 20 μm sagittal sections of perfusion-fixed, denervated and contralateral control hindpaws, by adapting standard hematoxylin and eosin (H&E) labeling protocols. Prior to labeling, tissue was snap-frozen in liquid nitrogen cooled isopentane, embedded in OCT, and sectioned on a cryostat [23].

### Statistics

Chi-square was performed to compare distributions of pressure ulcer formation between bandaged and unbandaged groups. Calculations were performed manually and *p*-values determined using a chi-square table for 3 degrees of freedom.

## Data Availability

The datasets during and/or analyzed during the current study available from the corresponding author on reasonable request.
